# Japanese pediatric and adult atomic bomb survivor dosimetry: potential improvements using the J45 phantom series and modern Monte Carlo transport

**DOI:** 10.1007/s00411-021-00946-2

**Published:** 2021-10-30

**Authors:** Keith T. Griffin, Tatsuhiko Sato, Sachiyo Funamoto, Konstantin Chizhov, Sean Domal, Colin Paulbeck, Wesley Bolch, Harry M. Cullings, Stephen Egbert, Akira Endo, Nolan Hertel, Choonsik Lee

**Affiliations:** 1 Radiation Epidemiology Branch, Division of Cancer Epidemiology and Genetics, National Cancer Institute, National Institutes of Health, 9609 Medical Center Drive, Rockville, MD 20850 USA; 2 Nuclear Science and Engineering Center, Japan Atomic Energy Agency, Tokai-mura, Japan; 3 Department of Statistics, Radiation Effects Research Foundation, Hiroshima, Japan; 4 J. Crayton Pruitt Family Department of Biomedical Engineering, University of Florida, Gainesville, FL USA; 5 San Diego, CA USA; 6 George W. Woodruff School of Mechanical Engineering, Georgia Institute of Technology, Atlanta, GA USA

**Keywords:** Atomic bomb survivors, Phantoms, Cohort dosimetry, Radiation transport, Phase space

## Abstract

The radiation exposure estimates for the atomic bomb survivors at Hiroshima and Nagasaki have evolved over the past several decades, reflecting a constant strive by the Radiation Effects Research Foundation (RERF) to provide thorough dosimetry to their cohort. Recently, a working group has introduced a new series of anatomical models, called the J45 phantom series, which improves upon those currently used at RERF through greater age resolution, sex distinction, anatomical realism, and organ dose availability. To evaluate the potential dosimetry improvements that would arise from their use in an RERF Dosimetry System, organ doses in the J45 series are evaluated here using environmental fluence data for 20 generalized survivor scenarios pulled directly from the current dosimetry system. The energy- and angle-dependent gamma and neutron fluences were converted to a source term for use in MCNP6, a modern Monte Carlo radiation transport code. Overall, the updated phantom series would be expected to provide dose improvements to several important organs, including the active marrow, colon, and stomach wall (up to 20, 20, and 15% impact on total dose, respectively). The impacts were especially significant for neutron dose estimates (up to a two-fold difference) and within organs which were unavailable in the previous phantom series. These impacts were consistent across the 20 scenarios and are potentially even greater when biological effectiveness of the neutron dose component is considered. The entirety of the dosimetry results for all organs are available as supplementary data, providing confident justification for potential future DS workflows utilizing the J45 phantom series.

**Supplementary Information:**

The online version contains supplementary material available at 10.1007/s00411-021-00946-2.

## Introduction

From decades of bi-national funding support, the Radiation Effects Research Foundation (RERF), and its predecessor, the Atomic Bomb Casualty Commission, have composed a detailed organ dosimetry system (DS) for the atomic bomb survivors at Hiroshima and Nagasaki, Japan. At RERF, radiation dose estimation constitutes a core research aim; this is because individual organ dose estimates are needed to define the relationships between radiation exposure and late-term health effects in the cohort of atomic bomb survivors. To date, the cohort managed by RERF remains one of the largest datasets including individualized organ dose estimates for an exposed population, with information on the atomic bomb radiation output and the subsequent transport through the environment including shielding structures, and the body on a case-by-case basis (Ozasa et al. 2018). Given the unique shielding environment experienced by each survivor, it follows that the dose estimates require an extensive dose reconstruction process. Careful and thorough record keeping on survivors’ details were taken by RERF, interviewing tens of thousands of survivors to detail multiple factors, including their physical location, orientation to the bomb, and shielding environment. Physical measurements from atomic testing projects and thermoluminescence analyses of material samples at Hiroshima and Nagasaki allowed RERF to accurately establish the bomb parameters. Simulation of the radiation transport was accomplished using discrete ordinate and adjoint Monte Carlo (MC) simulation codes. The workflow for all these methods to calculate individual survivor dose estimates is contained within their DS, which has been updated over multiple decades in a constant endeavor for improved dosimetry (Cullings et al. 2017; Roesch 1987; Young and Kerr 2005).

More recently, a working group comprised of members from Japanese and American research institutions has been established to study further improvements in RERF dosimetry through a contemporary update in the simulation of radiation transport through the body. Whereas RERF has previously utilized three simplified computational human models (phantoms) to represent the entire survivor cohort, a new series of phantoms has been produced by the working group based on body parameters of the 1945 Japanese population—thus called the J45 series. The J45 series provides many advantages: improvements in anatomical realism, improved distinction between body types based on age, sex, and pregnancy status, as well as a greater number of organs available for dosimetry than before. In their initial pilot studies, the working group used simplified planar source terms to irradiate the new J45 pediatric, adult, and pregnant phantom series and calculate the differences in organ dose coefficients expected between the old and new phantom series (Griffin et al. 2019; Paulbeck et al. 2019).

In the present work, angle- and energy-dependent gamma and neutron fluences have been taken directly from the DS database at RERF for 20 generalized survivor shielding scenarios; these fluences were converted into a phase space file usable within a modern MC code as a source term. The 20 shielding scenarios were simulated, with resulting organ dose determined for each survivor age and sex of the J45 pediatric and adult phantom series. Information on total particle fluences were used to convert per-particle dose reported by the MC code to an absolute dose received by the survivor. The organ doses estimated by the current DS for the generalized survivor were then compared to new results, showing dose differences based on both the use of a modern, forward MC radiation transport code and the use of updated phantom technology. Additionally, the current DS adult phantom was recreated in the modern MC code. Simulations using this phantom allowed distinction of the dose differences expected from the new phantom technology from those differences due to improvements in radiation transport simulation alone. These efforts will demonstrate how the potential implementation of the J45 series in a future RERF Dosimetry System could impact absolute survivor organ doses.

## Materials and methods

### RERF dosimetry systems methodology

At RERF, individualized survivor dose estimates are made through their DS workflow. Dosimetry System 1986 (DS86) was the first central dosimetry system at RERF to utilize computational simulations of radiation transport from the bomb source term through the human body to calculate organ dose (Roesch 1987). Dosimetry System 2002 (DS02) was their next and most recent core dosimetry system; it improved upon DS86 in many ways, including updates to the bomb source term, height of burst, radiation cross-sections for air, terrain shielding, house shielding refinements, fluence-to-KERMA factors (KERMA—kinetic energy released in matter), activation cross-sections, and fluence validation via physical measurements (Young and Kerr 2005). The most recent revision, DS02R1, reassessed individual survivor locations and terrain shielding data based on geographic information systems and corrections to survivor data (Cullings et al. 2017). Notably, most of the aspects related to radiation transport simulation have not been updated or modernized in nearly 35 years.

### Computational phantoms

**Fig. 1:**
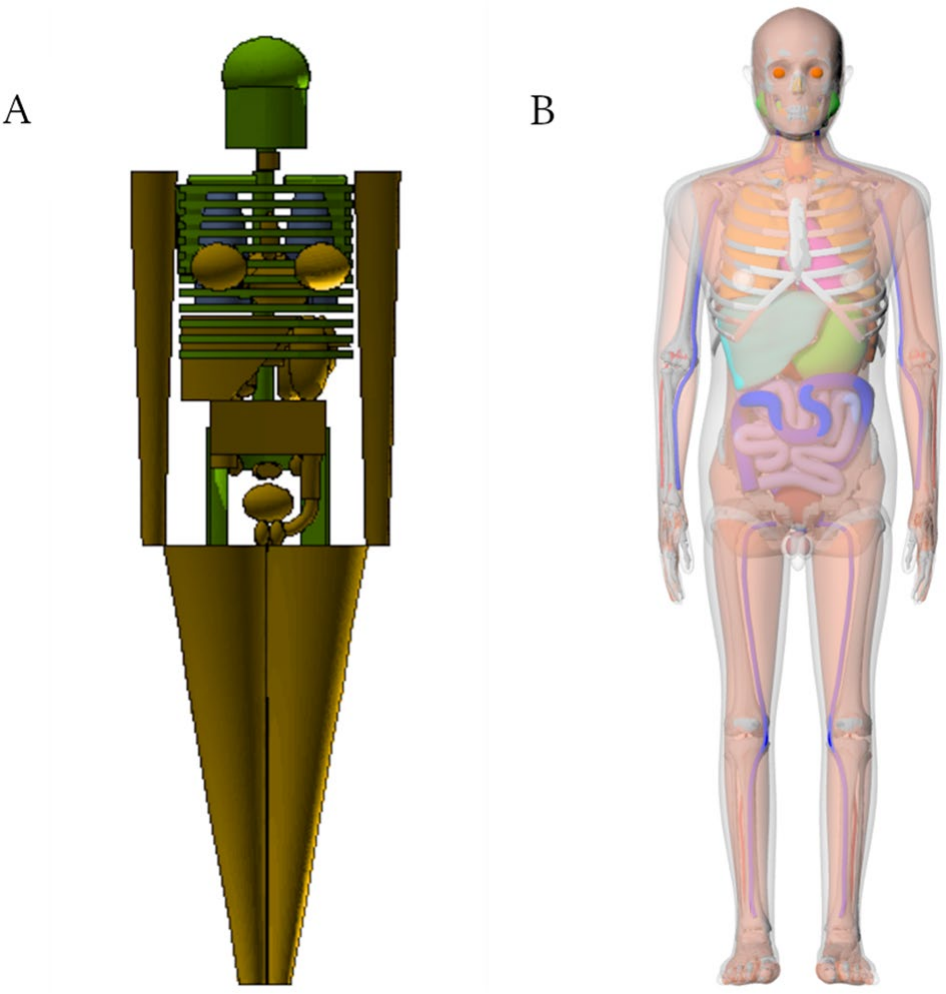
Three-dimensional visualization of the standing DS86/02 adult phantom with its trunk and head/neck soft tissue made transparent, recreated for use in forward Monte Carlo code (**A**) and the J45 adult male hybrid phantom (**B**)

The stylized phantoms developed for DS86 and re-used in DS02 (referred to here as the DS86/02 series) were based on the methodologies described by Eckerman et al. in their development of the Oak Ridge National Laboratory (ORNL) phantom series (Eckerman et al. 1996). The ORNL phantom series was stylized, meaning mathematically defined using a series of surface equations which collectively make up the body using Boolean operations to define organ volumes. In creating the DS86/02 series, these surface equations were adjusted to match known anatomical data on the Japanese population. Computational limitations of the past constrained RERF in their division of the population’s body size to only three age groupings: the infant, child, and adult. Within these groupings, the infant phantom represented survivors aged 0–2 years, the child represented survivors aged 3–11 years, and the adult represented survivors aged 12 years and beyond (shown in Fig. 1A). These phantoms were hermaphroditic, containing the female breasts and sex organs of both men and women.

### Calculation procedure

To compute organ doses for a specific survivor, RERF’s Dosimetry System relies on extensive databases on the radiation environment (i.e., particle fluences) at the two cities, which have been determined over the course of decades of research. From the initial bomb source term, air-over-ground radiation transport was conducted through a two-dimensional discrete ordinates simulation out to distances of 2,500 m at 25 m intervals, giving the free-in-air (in-open) angle- and energy-dependent particle (i.e., photon and neutron) fluences at these distances. Perturbations of these fluences by local environmental shielding, such as terrain and housing, were accounted for through adjoint Monte Carlo simulation. RERF categorized the housing and terrain shielding scenarios to create a fluence database for multiple locations within or outside of a model wooden house or tenement cluster. Inside the house, the shielding profile for a survivor was categorized via nine parameters, such as floor number and proximity of the person to a window, to create a “9-parameter” shielding model. Outside the house, an approach termed the GLOBE model was used, which uses portions of solid angle in azimuthal sections about the survivor that were estimated from early interview information about the structures surrounding each survivor; a survivor’s location inferred from street and terrain information is used to assign a position in the model housing cluster to obtain his or her shielded fluences. Thus, to encompass all cohort members, particle fluences were determined for both cities, at each interval of distance from the bomb hypocenter, and multiple categorized shielding situations. To determine organ dose for the survivor in these scenarios, the DS86/02 phantom series was placed inside of a coupling surface, with particle fluences expanded out to a spherical radius of 1 m surrounding the phantom. An adjoint MC code was then used to determine the organ fluences expected within the phantom by matching tracks that leave the organ to the expanded particle fluences at the spherical coupling surface. These organ fluences are finally translated into organ doses based on a soft tissue KERMA factor and a dose conversion factor for trabecular bone marrow. The Dosimetry System is thus capable of outputting both the particle fluences for a specific survivor shielding scenario, as well as his or her resultant doses, for use in the present study.

### Dosimetry system fluence and organ datasets

Throughout the workflow to perform survivor organ dosimetry, DS02 maintains a division of the total particle fluences into constituent primary and secondary particle fluences. Primary fluences include the prompt gammas and prompt neutrons (prompt emission from the bomb—within the first microseconds), as well as the delayed gammas and delayed neutrons (emission after some short time later by the nuclear reaction progeny); gammas generated within the air by these fluences were included within the primary gamma fluences. Secondary fluences include the gammas generated by interactions of the prompt and delayed neutrons in the survivor’s unique shielding environment (called the secondary shielded prompt neutron gammas and secondary shielded delayed neutron gammas). In total, six sets of fluence data are available from DS02 for each shielding scenario: the prompt neutron, delayed neutron, prompt gamma, delayed gamma, secondary prompt neutron gammas, and secondary delayed neutron gammas.

**Fig. 2:**
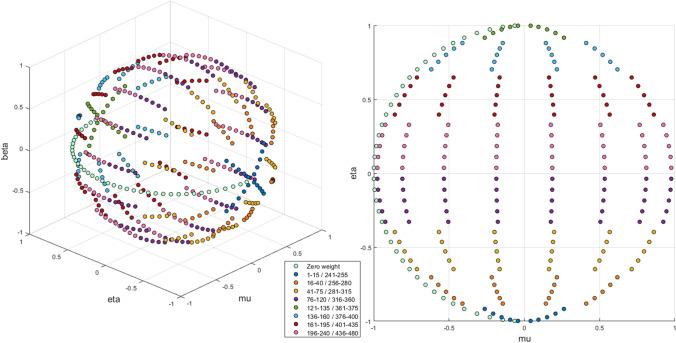
The 480 angular S-8 quadrature used to categorize the angular dependence of the photon and neutron fluences used within DS86 and DS02. Each angle bin can be uniquely described on the unit sphere by values of mu and eta, the direction cosines with respect to R and Z directions in cylindrical coordinates, as well as either a positive or negative value of beta. Angle bin numbers for the eight levels are provided within the legend. 48 angles were assigned zero weight (zero proportion of total solid angle)

The fluences from DS02 are double-differentially defined, in that they are both angle- and energy-dependent. Under most shielding scenarios, a survivor would experience asymmetric shielding from the bomb, and consequently the fluence is also asymmetric; however, for the unshielded, in-open scenario, symmetry in the angle-dependent fluence occurs, mirrored across the line extending outward from the hypocenter to the survivor. For both gammas and neutrons, the fluences are divided into either 240 directions (the symmetric, in-open scenario) or 480 directions (the asymmetric scenarios); these directions represent a derivative of S-8 symmetric angular quadrature for two-dimensional (R-Z) geometry, defining 48 angles over a 2π solid angle. This quadrature was customized by splitting every direction into five more directions to improve polar angular resolution – 240 directions for symmetric shielding and 480 angles for asymmetric shielding, with corresponding weights representing the angles’ fraction of total solid angle. However, eight of the original 48 angles were assigned zero weight in this quadrature to stabilize the discrete ordinates method; thus only 200 directions in symmetric and 400 directions in asymmetric shielding contribute to the fluence calculations. This S-8 quadrature is visualized within Fig. 2, with symmetry across the plane where beta is equal to zero. Within an angle bin, fluences are divided into 144 particle energy bins: 21 for each of the four categories of gammas, 37 for prompt neutrons, and 23 for delayed neutrons. More information on these quadrature and energy bins can be found in Table 3 in Appendix B of DS02 and in Table 6 in Chapter 12 of DS02, respectively (Young and Kerr 2005).

The resulting doses produced by DS02 are categorized by the six constituent sources, in addition to two further delineations: secondary gammas produced within the body via interactions of the prompt and delayed neutrons (called the body prompt/delayed neutron gammas). Organ doses are available from DS02 for: the brain, breasts, colon, eye lens, liver, lungs, ovaries, pancreas, stomach wall, testes, thyroid, urinary bladder wall, and uterus. Additionally, skeletal tissue doses are available for the active bone marrow (red bone marrow) and bone endosteum (bone surface). The maximum skin dose was also approximated by DS02 using the shielded air KERMA, which is the free-in-air KERMA after perturbation by any environmental shielding. All skin locations were assigned this dose.

## Recent improvements: organ dose working group methodology

### J45 phantom series

In Griffin et al. 2019, the working group developed a new series of hybrid phantoms based on the Japanese population of 1945, named the J45 series, which has greater anatomical realism and improved age resolution over those used previously by RERF (Griffin et al. 2019). Whereas the current DS86/02 three-member phantom series was stylized, the J45 series is part of the latest hybrid generation of phantoms, being composed of non-uniform rational b-spline (NURBS) surfaces and polygonal meshes; this allows detailed anatomical realism and flexibility through the support of computer aided design software. Phantoms at six ages were created: the newborn (0-year-old), 1-, 5-, 10-, 15-year-old, and adult (30-year-old). Sex-distinction in the phantom anatomy was also made, whereas the previous DS86/02 phantoms were hermaphroditic. The male and female phantoms at ages newborn to 15-years-old share nearly identical anatomy, with the exception of the sex-specific organs; the adult phantom anatomies vary in mass, height, and other anthropometric values, as given by Table 4 of DS86 Volume 1, Chapter 8. Multiple organs are newly available for dosimetry in the J45 series, as well as greater realism of the bone structure and up-to-date marrow distributions used in the active marrow and bone endosteum dosimetry. The adult male J45 phantom can be seen in Fig. 1B.

## Electron transport and air-generated electron phase space database

In the current DS, the gamma and neutron fluences calculated within an organ are converted to estimates of organ absorbed dose through the use of tissue fluence-to-KERMA factors, assuming all dose from the gammas and neutrons were locally delivered. However, the KERMA approximation is known to overestimate dose for external organs of the body, such as skin or the lens of the eye, where secondary generated electrons have enough range to leave the organ volume where they were generated (ICRP 2010). Updated calculations need to consider the transport of secondary electrons generated both within the body and from the surrounding air. Modern MC codes can efficiently handle the electron transport within the body. In previous work by Sato et al., development of an air-generated electron database was undertaken, providing a phase space of electrons generated outside of the body for the DS02 gamma fluences considered in the present work (Sato et al. 2020). The electrons arising from particle fluence interactions with 20 m of air were recorded onto the coupling surface surrounding the phantom; the distance of 20 m corresponds to the approximate range of a 4.5 MeV electron in air, which is expected to capture the majority of air electrons generated within the survivor’s local environment.

## Computational phantoms

The twelve-member J45 phantom series was used in the present work to represent the atomic bomb survivor cohort. These phantoms, which are originally in NURBS and polygonal mesh format, have previously been voxelized at resolutions ranging from 0.663 × 0.663 × 0.663 mm^3^ (newborn) to 1.579 × 1.579 × 2.207 mm^3^ (adult male) for use in MC code (Griffin et al. 2019). Additionally, the original DS86/02 phantom series was a potentially useful resource to the present study. Holding the simulation procedures constant, DS86/02 phantom dose results can provide information on the dose differences that are solely due to anatomical modelling improvements with the J45 series, and not from the update in simulation tools. However, the original DS86/02 phantoms, which were created for an older adjoint MC package, were not available in the format needed for present-day MC simulation. Fortunately, the DS86/02 phantom creation process is well-documented in Chapter 8, Appendix 1 of DS86 Volume 2 (Roesch 1987). Thus, reconstruction of the DS86/02 adult stylized phantom was possible through adjustment of the ORNL adult stylized phantom. Consequently, changes in the ORNL phantom surface equations were made to match those found in the DS86 report; this includes updates to the lungs, pancreas, thyroid, and testes volumes. The length of the legs was shortened to match the reported standing height of the 1945 Japanese adult. The head and neck regions were modified to match the style described in DS86, where a cylindrical neck region is defined containing the thyroid and the cervical spine. The arms were separated from the trunk of the body through bisection to make room for the conical frustums defining the arms. Bones within the arms and legs were defined with divisions into upper, middle, and lower portions to allow the use of the bone marrow fractions tabulated within DS86 for the active marrow and bone endosteum dosimetry. Lastly, the phantom materials and their elemental compositions were changed to fit into the three tissue categories used in the DS86/02 series: soft tissue, skeleton, and lung. This recreated DS86/02 adult phantom can be seen in Fig. 1a.

## Monte Carlo modelling

The Monte Carlo N-Particle version 6.1 (MCNP6) simulation package was used to model the radiation transport of the atomic bomb fluences onto the phantoms (Pelowitz 2013). Neutron cross-section data were retrieved from the ENDF/B-VI.6 library (U.S. Evaluated Nuclear Data File/B Version VI Release 6), with data tabulated at 293.6 K (Conlin et al, 2014). Neutron calculations within MCNP6 require specification of tissue composition in terms of elemental and isotopic fractions. When available, neutron cross-sections were used for elements composed of isotopes in their natural abundance; otherwise, cross-sections were specified for an element using the isotope of greatest natural abundance. Special treatment was given to molecularly bound hydrogen in light water to account for the thermal vibration of molecules, which is important for thermal neutron scattering cross-sections.

The radiation fields simulated within this work represent non-specific scenarios experienced by a survivor. Five generalized shielding scenarios were selected to cover the wide range of possible survivor shielding environments: in-open (IO), with no shielding; lightly-shielded within a “9-parameter” house (light 9p); heavily-shielded within a “9-parameter” house (heavy 9p); outside, in a lightly-shielded location using the GLOBE model (light glb); and outside, in a heavily-shielded location using the GLOBE model (heavy glb). Fluence data for a survivor under these shielding conditions was taken from DS02 for both cities and at distances of 1000 and 1500 m away from the hypocenter, resulting in 20 generalized shielding scenarios for this study. Within these shielding environments, the survivor was modelled to be facing towards the hypocenter.

## Fluence source terms

To create a simulation source term representative of the angle- and energy-dependent fluences produced by DS02, MATLAB code was created to generate an ASCII file containing a list of source particles. For each fluence type (e.g., prompt gamma, delayed neutron, etc.), a separate ASCII source term file was created. The relative probability of a particle starting with a particular energy or angle was determined according to the proportion of the particle fluence within an energy and angle bin to the total fluence of that emission type. Selection of an angle was chosen using probabilities based on the fluence in that angle bin, multiplied by that bin’s weight (proportion of total solid angle), out of the total weighted fluence. Within the angle bin, an energy bin was chosen based on the relative fraction of fluence in that energy bin out of the summed fluence across all energies for the angle. For gammas, the starting energy was chosen uniformly within the bin’s range; for neutrons, the energy was chosen based on the following criteria: at thermal energies, the neutron energy was chosen based on a 300° Maxwellian distribution; above thermal energies, the neutron energy was chosen with probability corresponding to inverse energy (1/E) dependence.

The starting particle’s location and direction are based exclusively on the angle bin selected for the particle. DS02 fluences are given for a point in air one meter above ground, and so this point fluence can be expanded outward to allow the placement of the phantom, with a vacuum media between the source and phantom to avoid double counting interactions in air. For newborns and one-year-old phantoms, the fluence was expanded to a radius of 40 cm; for five- and ten-year-old phantoms, the fluence was expanded to a radius of 70 cm; for fifteen-year-old and adult phantoms, the fluence was expanded to a radius of 100 cm. In the model, the surrounding spherical source was made as a composite of discs with similar radius as the sphere; each disc corresponds to one of the 400 angle bins from the S-8 expanded quadrature. Particle positions were first sampled uniformly upon a flat disc in the x–y plane of space. In S-8 quadrature, the different directions are represented by varying values of *mu* and *eta*, the direction cosines with respect to *R* and *Z* directions in cylindrical coordinates. These values were converted into a rotation of the flat disc in three-dimensional space—first, by rotation around the x-axis using the angle defined by *eta*, and second, by rotation around the z-axis using the angle defined by *mu*. Lastly, the disc was translated outwards in distance equal to the radius, opposite to the starting particles’ direction.

This process was used to create a log of ten million particles on each ASCII phase space file, where particle information is written line-by-line. MCNP6, however, does not accept ASCII phase space files—its phase space files are in binary format, with a header providing information on the model that generated it. To circumvent this issue, the free-to-use Monte Carlo Particle Lists (MCPL) distribution was used (Kittelmann et al. 2017). The MCPL distribution provides a set of C functions that allow the interchanging of particle state information between various MC codes. The distribution also includes C functions that allow the creation of intermediary MCPL files, which can be converted into functioning MCNP6 phase space files. For the present work, C +  + code was written to utilize these C functions to add particles from an ASCII file into a new MCPL file. Once created, the MCPL executable *mcpl2ssw* was used to convert the MCPL file to an MCNP phase space file. Since MCNP phase space format contains a unique header based on the simulation that created it, the MCPL *mcpl2ssw* function also requires a reference MCNP phase space file to mimic the header. It was found that by retaining one dummy surface in both the input file used to create a reference phase space file and the actual simulation input files, the phase space file header was appropriately configured by MCPL. The MCNP phase space format requires that starting particles be assigned a surface to start on; however, no errors were thrown by MCNP6 for assigning the dummy surface to a particle and then starting that particle outside of that surface. The resulting MCNP6 phase space files were verified through the use of the PTRAC card, allowing the output of the simulation to include an ASCII file containing the position, direction, and energy of all source particles in the problem. Comparisons of source particles between the initial ASCII files and the PTRAC output generated by MCNP6 were found to match.

### Dose tallying

The phase space source terms created through this work, as well as the air-generated electron database created by Sato et al. (2020), were used to calculate organ absorbed doses within the J45 phantom series for 28 organs and tissues: the adrenal glands, brain, breasts, colon, esophagus, extrathoracic region, eye lens, gall bladder wall, heart wall, kidneys, liver, lungs, lymph nodes, muscle, oral mucosa, ovaries, pancreas, prostate, salivary glands, small intestine wall, skin, spleen, stomach wall, testes, thymus, thyroid, urinary bladder wall, and uterus. For simulations of the neutron spectra, the F6 tally (MeV/g) was used to record neutron organ dose deposition. For simulations of the gamma spectra and air-generated electrons, the *F8 tally (MeV) was used to record organ dose deposition; additionally, in simulations of the neutron spectra, the *F8 tally was utilized to record dose delivered by gammas produced within the body (reported as body prompt/delayed neutron gammas). Active bone marrow and bone endosteum doses were also calculated using fluence-to-dose response functions developed at the University of Florida (Bahadori et al. 2011; Johnson et al., 2011). By recording the neutron and gamma fluence at 25 bone sites using the F4 tally (1/cm^2^), the tabulated response functions allow conversion to dose at each bone site. Age-dependent bone marrow distribution fractions were then used with each bone site’s dose estimate to make a weighted-sum estimate of the total active marrow and bone endosteum doses. Results from MCNP6 were converted from per-particle dose to an absolute dose for the survivor through multiplication with the total particle fluence.

### Statistical uncertainties

In total, 2208 MCNP6 simulations were run for the J45 series, based on the 20 survivor scenarios, six subdivided spectra and associated air electron simulations, and twelve J45 voxel phantoms. Each neutron and gamma simulation ran between 500 million and 1 billion particles sampled from the phase space file, respectively; air-generated electron simulations ran 20 million particles. Although only 10 million particles were written to the phase space files, MCNP6 resamples from these particles with a different starting seed to improve statistics. All simulations were run on the BioWulf high-performance computing system at the NIH, using a Linux computing cluster with typical nodes consisting of two central processing units (CPUs) with 16 threads each. In this environment, each simulation took between 3 and 10 h to complete, resulting in nearly 150,000 CPU-hours dedicated to this task. For the roughly 62,000 doses estimated via MCNP6, nearly all were made to within ± 5% MC statistical error; the main exception was in the eye lens dose and breast dose (at young ages), with error to within ± 20%, and air electron doses for internal organs, for which the doses are negligible. When the organ doses were combined across all fluence types, the propagated errors in total dose estimates were to within ± 3%, except for total dose of the eye lens and breasts, which were to within ± 10%.

## Organ surrogacy for dose response risk assessments

Previous use of the DS86/02 stylized phantom series restricted organ dose calculations to a subset of organs of interest to epidemiologists. When determining the dose–response relationship for a disease in an undefined organ in the DS86/02 stylized phantom series, the dose to a similar surrogate organ had to be used instead. Given the greater availability of organs within the new J45 voxel phantom series, this work aimed to quantify how a phantom update may impact the dosimetry for risk estimates that utilize surrogate organs. Comparisons between the DS02 surrogate dose and the newly-available J45 true organ dose can accomplish this task. For such surrogate pairs described in Preston et al. (Preston et al. 2007), the impacts on dosimetry were determined (undefined organ and surrogate): extrathoracic region and skin; prostate and urinary bladder wall; gallbladder wall and pancreas; and esophagus and stomach wall. Similarly, studies on melanoma and nonmelanoma cancer utilize skin dose, which was not an available organ in the DS86/02 phantom (Ron et al. 1998; Sugiyama et al. 2014); instead, DS02 reports shielded air KERMA as the surrogate dose. This shielded air KERMA from DS02 was thus compared to the dose from the skin organ in the J45 phantom series to determine the potential impact on skin dose estimates from the phantom update.

## Results and discussion

The results for this study are broken down into expected differences from the new phantom series and those differences due to the use of a modern MC radiation transport code. Additionally, the effects of organ surrogacy will be discussed. Figures show the breakdown in total organ dose to the dose from constituent particle fluences using stacked bar graphs. The summation of stacked bars underneath prompt neutron (green) indicates the total gamma dose, the major component of dose to the survivor. In the tables with color visualization, deeper red hues represent greater dose underestimation by DS02, while deeper blue hues represent greater dose overestimation by DS02 (as compared to J45 dose). The resulting organ dose differences will mostly be compared here for organs with the greatest radiation protection tissue weighting factors – the active marrow, breasts, colon, lungs, and stomach wall (ICRP 2007). They will also mostly be discussed for Hiroshima, in-open, at 1000 m from hypocenter. The entire dataset of calculated organ doses and their comparison to DS02 data can be found in the online supplement.

## Impact of Monte Carlo update

**Fig. 3:**
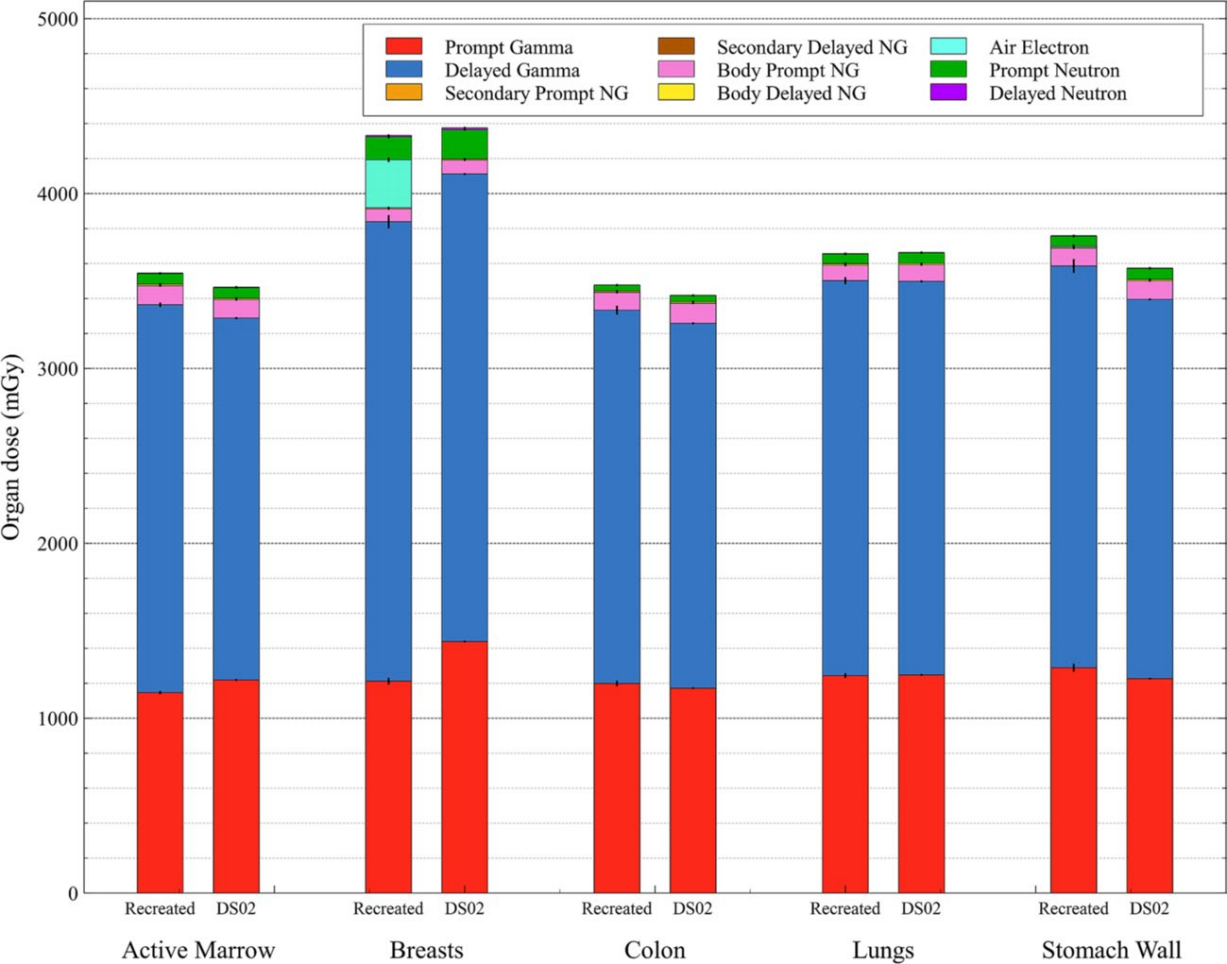
Comparisons of adult organ dose estimates taken from DS02 to those calculated in the present study by MCNP6 using the recreated DS86/02 adult phantom. Dose is broken down into nine components from different particles fluences for survivors at Hiroshima, in-open, at 1000 m from hypocenter. Black lines represent the associated Monte Carlo error of the stacked bar underneath; NG – neutron-induced gammas

In Fig. 3, dose comparison results between the recreated DS86/02 phantom doses calculated by means of MCNP6 and the doses taken directly from DS02 are shown for the active marrow, breasts, colon, lungs, and stomach wall. In Table 1, the percent differences in total dose, total neutron dose, and total gamma dose are given for these organs. Notably, both methodologies had resulting total dose and total gamma dose estimates to within about 5% of one another. The neutron doses, on the other hand, show slightly greater variability. These differences may be due to updates in the neutron transport within the codes (e.g., cross-section differences) as well as differences due to dose tallying (e.g., using DS02 fluence-to-KERMA conversion coefficients vs. the MCNP6 F6 heating tally).

**Table 1:** Percent difference in estimated organ dose for an adult survivor at Hiroshima, in-open, 1000 m from hypocenter, between modern simulation results in MCNP6 using the recreated DS02 adult phantom and those results pulled directly from DS02

Organ	Dose category	DS02 recreated	% Diff	DS02 results
Active marrow^a^	Total dose (mGy)	3545.2	− 2.3%	3465.3
	Neutron dose	62.5	1.9%	63.6
	Gamma dose	3482.7	− 2.3%	3401.7
Breasts	Total dose (mGy)	4332.6	1.0%	4375.8
	Neutron dose	138.8	28.1%	177.8
	Gamma dose	4193.8	0.1%	4198.0
Colon	Total dose (mGy)	3477.3	−1.7%	3418.3
	Neutron dose	33.2	9.1%	36.3
	Gamma dose	3444.1	− 1.8%	3382.0
Lungs	Total dose (mGy)	3657.0	0.2%	3663.5
	Neutron dose	56.2	11.9%	62.9
	Gamma dose	3600.8	0.0%	3600.6
Stomach Wall	Total dose (mGy)	3759.1	− 4.9%	3574.2
	Neutron dose	56.3	14.8%	64.6
	Gamma dose	3702.8	− 5.2%	3509.6

Neutron dose is unweighted by any potential relative biological effectiveness (RBE) in the total dose

^a^Active marrow dose calculations in the recreated phantom followed the methodology described within DS02, not using the up-to-date bone marrow fractions or dose–response functions used for the J45 phantom series

From the results of Table 1, it is anticipated that the potential impacts from an update in survivor dosimetry using the J45 series would mostly be due to the new phantom anatomies and not the improvements in radiation transport simulation itself. In the active marrow calculations, dose was relatively unaffected by the use of a modern transport code; as will be shown below, however, significant differences were seen in the estimated active marrow neutron dose from the use of the J45 phantom series. This is likely due to the J45 series’ updated neutron fluence-to-dose response functions and marrow distribution fractions. A similar assessment can be made for the colon and stomach wall. Though the impacts to total dose were small when using the recreated phantom, these organ doses were significantly different when using the J45 series. This may be attributed to organs being positioned either more forwardly (colon) or more deeply (stomach wall) within the body of the J45 adult than in the DS86/02 adult phantom. Previous work by the authors has suggested that neutron dose in survivor dosimetry is more sensitive to organ positioning (and thus body self-shielding) than gamma dose (Griffin et al. 2019; Paulbeck et al. 2019).

## Impact of phantom update

### Age classification

**Fig. 4:**
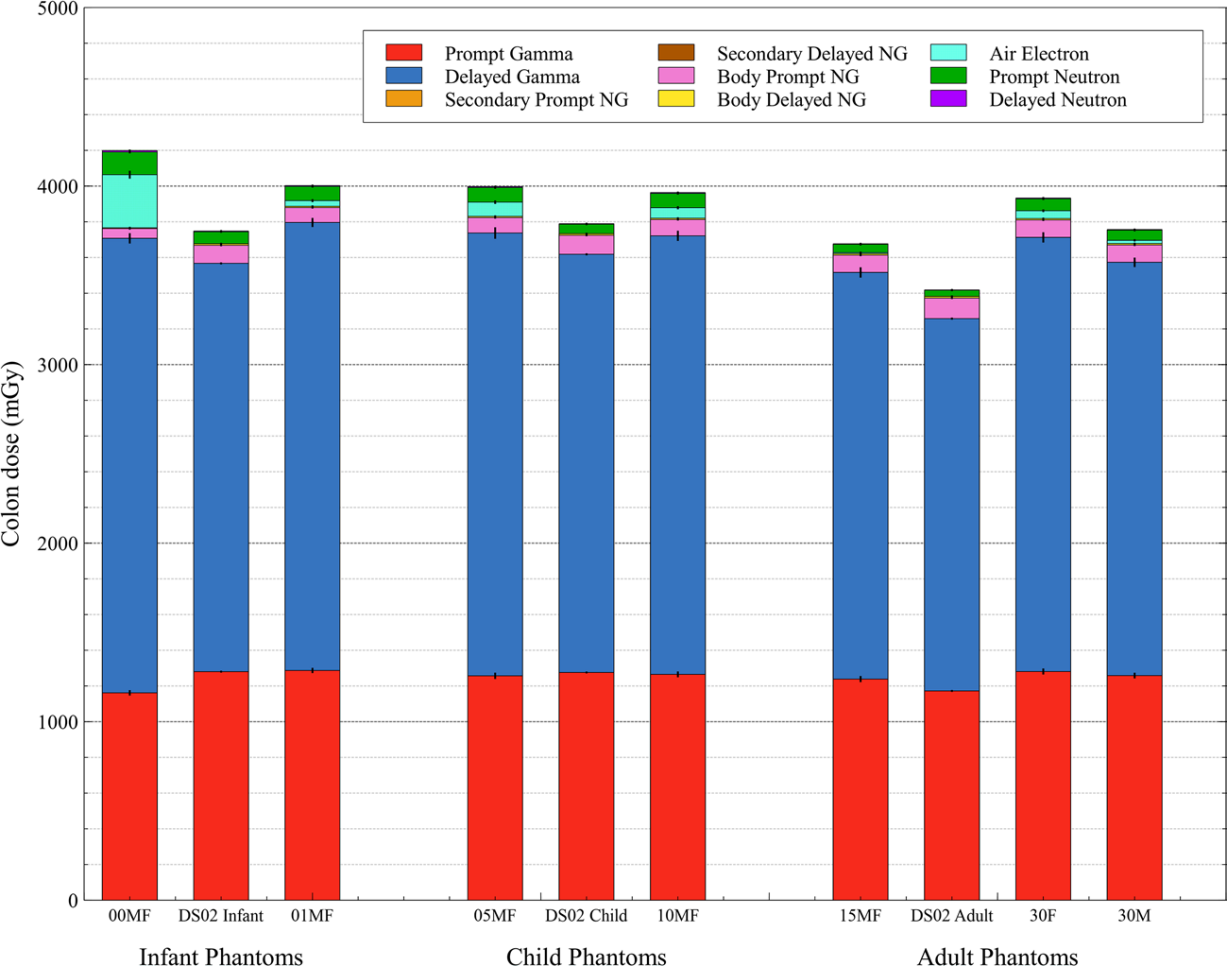
Comparisons of colon dose estimates taken from DS02 to those calculated within MCNP6 using the J45 voxel phantom series within each of the three age classifications (nomenclature: 00, 01, 05, 10, 15, and 30 is the J45 phantom age; M/F (male/female) is the phantom sex). Dose is broken down into nine components (NG are neutron-induced gammas) from different particle fluences for survivors at Hiroshima, in-open shielding, at 1000 m from hypocenter. Black lines represent the associated Monte Carlo error of the stacked bar underneath

Figure 4 provides a comparison of colon dose estimates across multiple survivor ages for the doses calculated within this study using the J45 voxel phantom series to those taken directly from DS02. Table 2 tabulates the percent differences in colon dose at these age groups, as well as for active marrow, breasts, lungs, and stomach wall dose. Across all age groups, it can be noted that the total colon dose was comparatively underestimated by the DS02 methodology. Within the infant classification, the underestimation by DS02 was greater for the infant survivor than for the one-year-old survivor. This underestimation seems to be driven by the much greater gamma dose found using electron transport within the J45 series. The J45 infant has a significant portion of dose arising from electrons generated within the air, which are able to penetrate to the colon due to the small amount of frontal tissue shielding at this age. Some of the colon dose underestimation at younger ages can thus be attributed to the previous use of the KERMA approximation in DS02. Within the child classification, a similar underestimation of total colon dose is seen for both the five-year and ten-year-old survivors. Within the adult classification, the adult female survivor’s total colon dose was the most underestimated of all survivors – roughly 13%. A quick analysis of the colon’s frontal shielding in both the J45 adult female and recreated DS86/02 adult phantom was performed, using methods published previously (Griffin et al. 2020). It was found that the colon is positioned much more forwardly within the body of the J45 adults than in the DS86/02 adult phantom (a mean distance of 4.1 cm closer to the front of the body); a comparison plot of the organ depth distributions may be found in the supplementary data. For this study, where the survivor faces hypocenter, the amount of frontal shielding for the colon greatly affects its dosimetry, but this effect may be less significant for other survivor orientations.

**Table 2:**
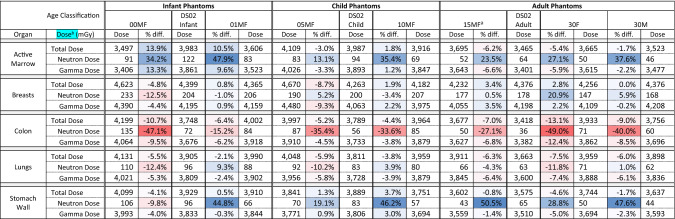
Percent difference in organ dose estimates by age classification between the J45 series and DS02 results, for survivors at Hiroshima, in-open, 1000 m from hypocenter

Deeper red hues represent greater dose underestimation by DS02, while deeper blue hues represent greater dose overestimation by DS02 (as compared to J45 dose). Air electron dose for the J45 series is reported under Gamma Dose. Neutron dose is unweighted by any potential relative biological effectiveness (RBE) in the total dose. For these results, the male and female J45 phantoms share nearly identical dose results up through age 15, except for the breasts

^a^Results for the 15-year-old J45 breasts are given for the female, comparable to the female breasts of the hermaphroditic DS02 adult phantom

^b^Dose results are truncated at the decimal point. Percent difference values are accurate as reported to the first decimal point

Looking at Table 2, the five organs shown are not expected to have total dose change for a given survivor by more than 20%; this is also true of nearly all the organs calculated and compared by this study (see supplementary data). The active marrow total dose estimates were not significantly impacted by the use of the J45 voxel series, except for the infant ages which showed a roughly 10–20% overestimation by DS02. However, DS02 showed significant overestimates of neutron dose in the active marrow compared to J45 results. This is due to multiple factors, including: the new anatomical bone definitions, the updated active marrow fractions at each bone site, and the updated neutron fluence-to-dose response functions at each bone site. Breast dose was found to be minimally impacted by the shift to J45 phantoms, except in neutron dose estimation for the adult female survivor. Lung dose was also found to be minimally impacted, with a maximum difference in total lung dose seen for the adult female at 7.5%. Colon dose differences shown in Table 2 depict strong evidence of neutron dose underestimation by DS02 for all ages. In contrast, percent differences for stomach wall dose estimates show strong evidence of neutron dose overestimation by DS02 for all ages except the infant.

Gamma dose is the dominant component of the total dose received by a survivor; this remains true for all survivor scenarios observed in this study and is especially true at Nagasaki. Though the neutron component of total dose has been shown to drastically change in some organs due to the phantom update, its effect on total dose differences is minimal due to the small proportional contribution of the neutron absorbed dose to the total absorbed dose. However, in epidemiological studies involved with the Life Span Study, neutron dose is often weighted by a factor of 10 to account for a neutron’s greater relative biological effectiveness (RBE) (Grant et al. 2017; Preston et al. 2007). This number is not set in stone; data-driven approaches have previously been made to determine neutron RBE values using Life Span Study data on neutron and gamma dose in combination with excess cancer risks; Cordova and Cullings found that the neutron RBE for the colon may be up to a value of 80 (Cordova and Cullings 2019). This would make neutron dose improvements from the J45 series much more significant, both for studies using and determining neutron RBE. For instance, the future use of a neutron RBE weighting of either 10 or 80 would cause the impact to total colon dose seen in Table 2 for the adult female to rise from 13% to roughly 18% and 34%, respectively.

The results described thus far have been for the shielding scenario of a survivor, in-open at 1000 m from hypocenter at Hiroshima; they have not yet shown how these results hold up under the other shielding scenarios considered by this work. Across the 20 survivor scenarios, Table 3 provides a comparison of percent differences in adult male survivor doses between J45 and DS02 for the active marrow, colon, and lungs. Note that percent differences in total dose do not vary more than about 8% depending on the shielding scenario. Thus, most of the trends in total dose described for the shielding scenarios discussed here also apply for other shielding scenarios experienced by survivors. Percent changes in neutron dose are shown to vary up to 20% for both the active marrow and colon, depending on the shielding scenario. Also note that neutron dose differences between the J45 series and DS02 are typically smaller for greater distances from hypocenter. This is likely due to hardening of the neutron spectra with greater attenuation through air; with harder spectra, differences in phantom modelling make less impact on organ dosimetry.

**Table 3:**
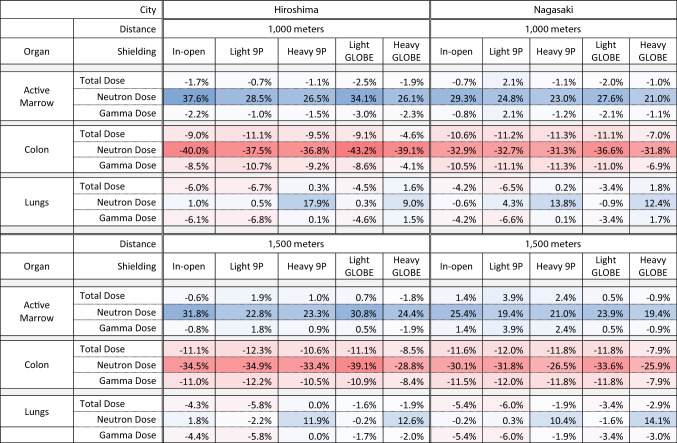
Percent difference in organ dose estimates between the J45 series and DS02 results for the adult male survivor across the 20 survivor shielding scenarios considered by this work

Deeper red hues represent greater dose underestimation by DS02, while deeper blue hues represent greater dose overestimation by DS02 (as compared to J45 dose). Air electron dose for the J45 series is reported under Gamma Dose. Neutron dose is unweighted by any potential relative biological effectiveness (RBE) in the total dose

### Organ surrogacy

**Fig. 5:**
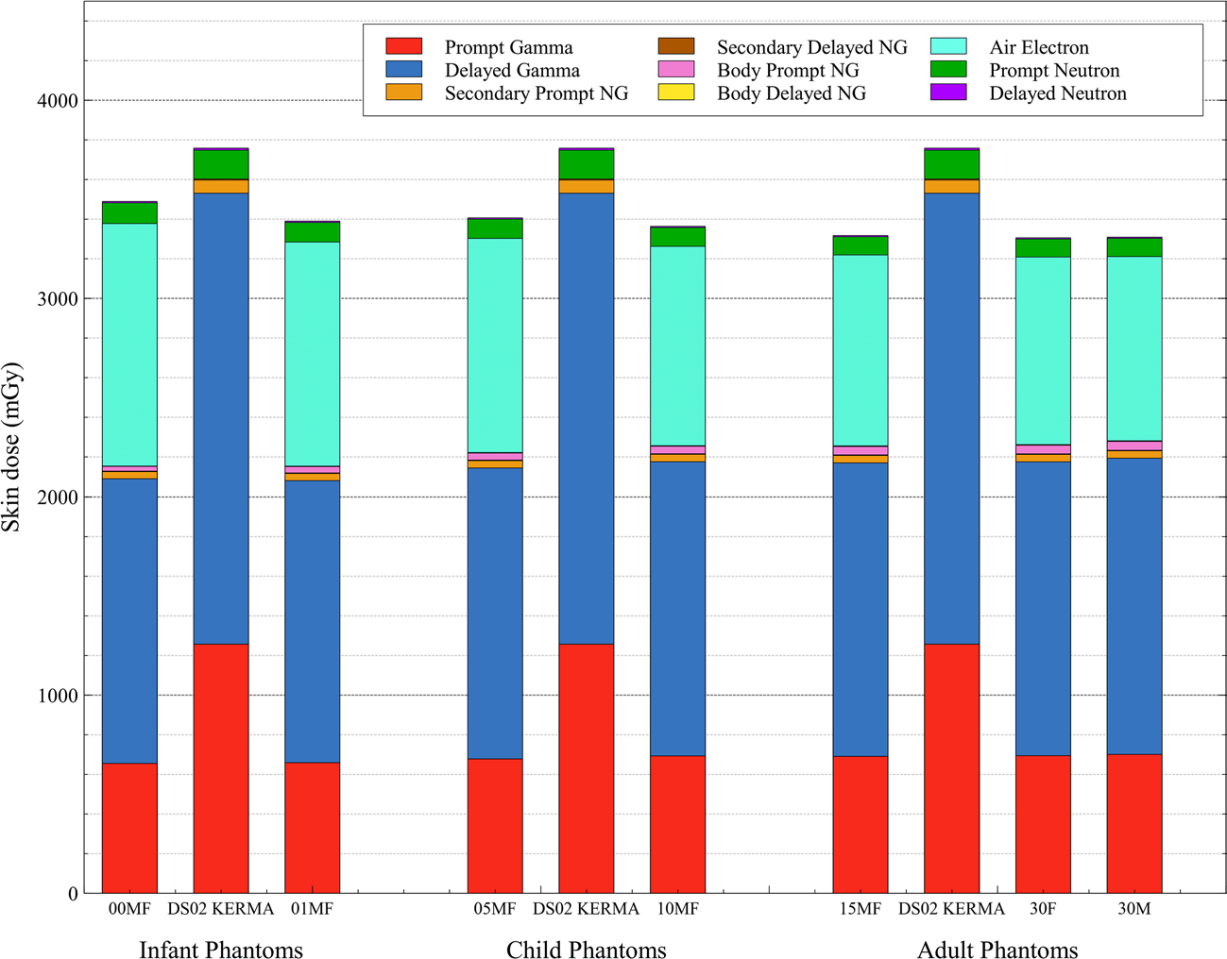
Comparisons of skin dose estimates taken from DS02, which used shielded air KERMA as a surrogate, to those calculated within MCNP6 using the J45 voxel series within each of the three age classifications (nomenclature: 00, 01, 05, 10, 15, and 30 is the J45 phantom age; M/F (male/female) is the phantom sex). Dose is broken down into nine components (NG are neutron-induced gammas) from different particle fluences for survivors at Hiroshima, under light 9p shielding, at 1000 m from hypocenter. Error bars are not displayed, due to insignificant magnitude

With more organs available in the J45 series, this section discusses improvements in organ dose estimates given that surrogate doses would no longer be needed. In Fig. 5, skin dose estimates in the new J45 series are compared to the shielded air KERMA surrogate dose used by DS02. Note that the DS02 skin dose surrogate estimate is constant across all ages, as the DS02 phantom is not used in the calculation of air KERMA. From this figure, the significant overestimate of total skin dose can be seen. This result was expected, as the shielded air KERMA surrogacy had originally been chosen to signify the maximum skin dose on the body, regardless of orientation to the hypocenter or posture of the phantom. The self-shielding provided for the skin on the opposite side of the body to incident radiation decreases the averaged skin dose estimate in the J45 series. Overestimation by DS02 can be seen for both gamma and neutron dose. The greatest skin dose overestimation in Fig. 5 is seen for the adult survivors, with total gamma and total neutron dose differences of 12 and 59%, respectively.

In Table 4, the percent difference in surrogate dose (from DS02) to dose now available in J45 series is evaluated for four organs not found in the DS02 phantom: the esophagus, gallbladder, prostate, and the extrathoracic airways. The surrogate organ doses used in risk estimates for these studies were the stomach wall, pancreas, urinary bladder wall, and skin (shielded air KERMA), respectively. In terms of total dose, it was found that the surrogate organs were somewhat reasonable in their estimation of the actual organ’s dose, with percent differences within 15% for all ages. Once again, it is noted that neutron dose shows the greatest percent difference. For the esophagus and extrathoracic airways, the DS02 surrogate doses showed a consistent overestimation of the actual organ’s neutron dose, in some cases by a factor of two or more. In previous work, it was shown that the skin was a poor surrogate for extrathoracic airway dose, even when comparing J45 skin dose to J45 extrathoracic dose (Griffin et al. 2019). This overestimation is magnified when utilizing the shielded air KERMA from DS02 to estimate the extrathoracic dose, which explains the large differences seen. The previous work also predicted the results seen for the esophagus, likely due to differences in body shielding between the esophagus and the surrogate stomach wall. Results for the gallbladder and prostate showed small, expected changes in dose estimates when using the newly available organs in the J45 series, with some large differences possible within the infant age classification.

**Table 4:**
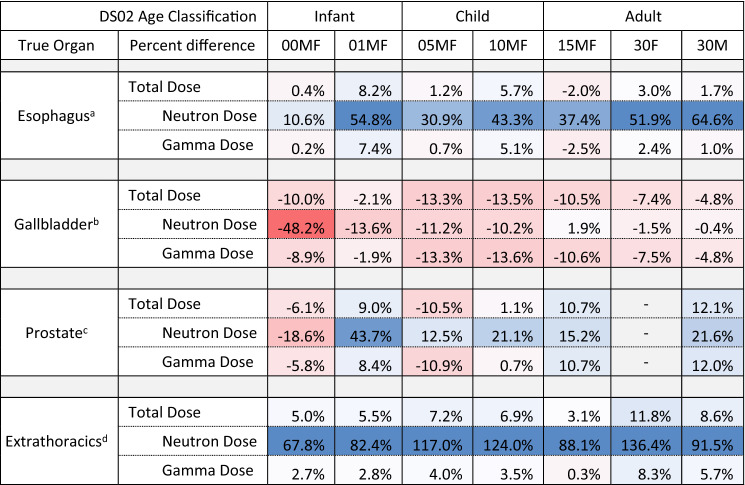
Percent difference in organ dose estimates between the J45 actual organ dose and the dose taken from DS02 for the epidemiological surrogate (see footnote), in-open, at Hiroshima 1000 m from hypocenter

Deeper red hues represent greater dose underestimation by DS02, while deeper blue hues represent greater dose overestimation by DS02 (as compared to J45 dose). Neutron dose is unweighted by any potential relative biological effectiveness (RBE) in the total dose

Given these four organ doses were not estimated by DS02, the following surrogate organ doses have previously been used in epidemiological studies: ^a^Stomach wall, ^b^Pancreas, ^c^Urinary bladder wall, and ^d^Skin (shielded air KERMA)

The colon organ has historically been used as a comprehensive surrogate organ in the Life Span Study, vital for applications that require a single dose in the risk analysis, such as the “all solid cancer” endpoint (Grant et al. 2017; Preston et al. 2007). Evaluation of the colon organ as a comprehensive surrogate had previously been performed by the authors and was once again briefly examined within this work (Griffin et al. 2019). In these investigations, organ doses within the J45 series were compared to one another to determine which body organ is most representative of all the rest; these results are available in the supplementary data. Unfortunately, there is no clear winner as to which organ dose works best as the comprehensive surrogate. Given the wide variety of organ shapes and positions within the body, one should not expect a single organ dose to be purely representative of all other organ doses in the body. In the future, a radiation dose index may perform better for studies on aggregate cancer risks, one which is created as a sum of organ doses weighted by each organ’s proportion of radiation-linked cancers (excess relative risk greater than 0) falling within the group.

## Study limitations

While the results described thus far for the J45 series indicate substantial potential improvements to the dosimetry performed at RERF, there are some limitations that should be addressed. First, the results shown in this work are limited to standing individuals facing towards hypocenter; the results do not cover the many other potential positions of the cohort members, including kneeling—a position nearly one third of the cohort was oriented in at the time of exposure. At Nagasaki, there were also a significant number of cohort members who were working in a processing factory, which is a unique shielding environment due to the workbenches and building materials. Exceptional scenarios such as those will be confronted in future studies by the working group.

Additionally, computational time would be a limiting factor to the application of the applied methods in a future dosimetry system. The scope of the present work only considered a generalized subset of cohort members for two distances from hypocenter; this effort alone took nearly 150,000 CPU-hours. Continuing these simulations for the many other survivor scenarios (e.g., different orientations to hypocenter, other distances from hypocenter, specific environment shielding, etc.), would require an impractical amount of time spent using resources on computing clusters. Instead, the working group has devoted future efforts to a more modular approach: calculating dose response functions in the J45 series for the particle fluences produced by RERF, which are pre-calculated and binned by angle and energy. For each organ in the J45 series, as well as each energy and angle for a gamma or neutron source, a dose response function will be developed to convert fluence to dose. Thus, one may potentially utilize such a library of response functions to build survivor doses based on the available particle fluences at each survivor’s specific environment. More information on this process can be found in Sato et al. (2020).

## Conclusions

A new series of phantoms produced by a multi-institutional working group (the J45 phantom series) has been shown to provide multiple advantages in survivor dosimetry for the atomic bomb survivors’ cohort. In the present work, the neutron and gamma fluences for 20 generalized survivor scenarios at Hiroshima and Nagasaki were taken from the DS database at RERF and used as a source term in modern MC code simulations. Through this effort, an accurate portrayal of the absolute organ dose differences between DS02 and new MC results was demonstrated, showing differences based on both the use of a state-of-the-art MC radiation transport code and the use of updated phantom technology. Overall, the impacts of new phantom ages, sex-distinction, more available organs, and detailed anatomy were found to be significant for multiple organs, including the active marrow, colon, and stomach wall. These impacts were consistent across the 20 survivor shielding scenarios considered by this work and are potentially greater when RBE weighting of the neutron dose component is considered. On the other hand, the impacts from modern updates to MC radiation transport were found to be less important. The entirety of the results calculated as part of this study are tabulated and available as supplementary data online, providing confident justification for a potential future DS workflow utilizing the J45 phantom series.

## Supplementary Information

Below is the link to the electronic supplementary material.

Supplementary file1 (ZIP 2974 KB)

## Data Availability

The organ doses calculated within this study for the J45 phantom series, as well as a more comprehensive set of comparisons to DS02 organ doses, are provided through the supplementary information online. One figure is also provided in the supplementary information, depicting the difference in colon depth below the body surface between the DS02 adult phantom and the J45 adult female phantom.
