# Supplementary figures and images for: Japanese pediatric and adult atomic bomb survivor dosimetry: potential improvements using the J45 phantom series and modern Monte Carlo transport
Source: Radiat Environ Biophys. 2021 Oct 30;61(1):73–86. doi: 10.1007/s00411-021-00946-2 (PMC8897329; doi:10.1007/s00411-021-00946-2)

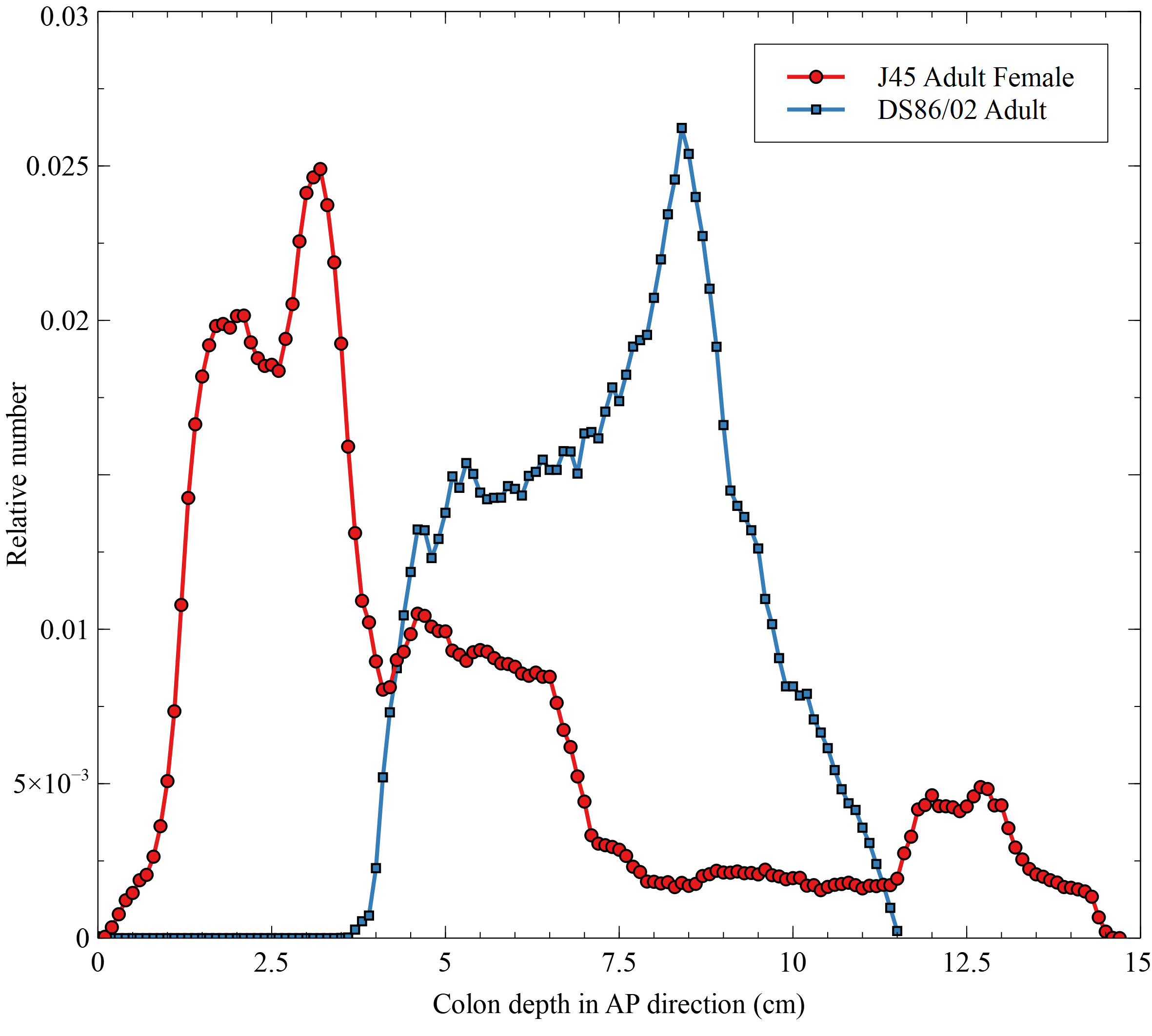

Supplement: Supplementary file 1 — Supplementary file1 (ZIP 2974 KB) [file 411_2021_946_MOESM1_ESM.zip › _supplementary/colon_depth_ADf_AP.png]
